# Cytocompatibility and Bioactive Properties of Hydraulic Calcium Silicate-Based Cements (HCSCs) on Stem Cells from Human Exfoliated Deciduous Teeth (SHEDs): A Systematic Review of In Vitro Studies

**DOI:** 10.3390/jcm9123872

**Published:** 2020-11-28

**Authors:** José Luis Sanz, Leopoldo Forner, Carmen Llena, Julia Guerrero-Gironés, María Melo, Sandro Rengo, Gianrico Spagnuolo, Francisco Javier Rodríguez-Lozano

**Affiliations:** 1 Departament d’Estomatologia, Facultat de Medicina I Odontologia, Universitat de València, 46010 Valencia, Spain; jsanzalex96@gmail.com (J.L.S.); llena@uv.es (C.L.); m.pilar.melo@uv.es (M.M.); 2 Cellular Therapy and Hematopoietic Transplant Research Group, Biomedical Research Institute, Virgen de la Arrixaca Clinical University Hospital, IMIB-Arrixaca, University of Murcia, 30120 Murcia, Spain; juliaguerrero1@hotmail.com (J.G.-G.); fcojavier@um.es (F.J.R.-L.); 3 Department of Dermatology, Stomatology, Radiology and Physical Medicine, Morales Meseguer Hospital, Faculty of Medicine, University of Murcia, 30100 Murcia, Spain; 4 Department of Neurosciences, Reproductive and Odontostomatological Sciences, University of Naples “Federico II”, 80138 Napoli, Italy; sanrengo@unina.it (S.R.); gspagnuo@unina.it (G.S.); 5 Institute of Dentistry, I. M. Sechenov First Moscow State Medical University, Moscow 119146, Russia

**Keywords:** calcium silicate cements, bioceramic, dental stem cells, cell viability, bioactivity

## Abstract

The implementation of hydraulic calcium silicate-based endodontic cements (HCSCs) in biologically based endodontic procedures for the primary dentition has been recently investigated, focusing on the biological response of stem cells from human exfoliated deciduous teeth (SHEDs) towards them. The present systematic review aimed to present a qualitative synthesis of the available literature consisting of in vitro assays, which assessed the cytocompatibility and bioactive properties of HCSCs in direct contact with SHEDs. Following the PRISMA statement, an electronic database search was carried out in Medline, Scopus, Embase, Web of Science, and SciELO on March 31st and updated on November 16th, 2020. In vitro studies evaluating the biological response of SHEDs to the treatment with HCSCs were eligible. Within the term biological response, assays assessing the cytocompatibility (i.e., cell viability, migration, proliferation), cell plasticity or differentiation (i.e., osteo/odontogenic marker expression), and bioactivity or biomineralization (i.e., mineralized nodule formation) were included. A total of seven studies were included after the selection process. The study sample comprised an extensive range of cell viability, migration, proliferation, adhesion, and bioactivity assays regarding the biological response of SHEDs towards five different commercially available HCSCs (MTA, ProRoot MTA, Biodentine, iRoot BP Plus, and Theracal LC). Biodentine, MTA, and iRoot BP Plus showed significant positive results in cytocompatibility and bioactivity assays when cultured with SHEDs. The results from in vitro assays assessing the cytocompatibility and bioactivity of the HCSCs MTA, Biodentine, and iRoot BP Plus towards SHEDs support their use in vital pulp treatment for the primary dentition.

## 1. Introduction

The preservation of primary or deciduous teeth until their physiological exfoliation is essential for the correct development of the dental arches, the maxillae, and eruption of the permanent dentition [1]. Dental trauma and carious lesions may act as potential factors for the premature loss of primary teeth. Both of these factors, depending on their severity, extension, and evolution, may damage the dentin-pulp complex and induce a subsequent inflammatory and reparative response from the affected tissue [2].

This response is encompassed within the term reparative dentinogenesis, a physiological process of tissue neoformation, which involves an intricate interrelation of molecular signaling cascades [3]. In turn, it has been posed that this mechanism results in the differentiation of mesenchymal cells of dental origin or dental stem cells (DSCs) into odontoblast-like cells, which mediate the formation of new tissue with similar characteristics [4]. Various stem cell phenotypes have been isolated and characterized within the group of DSCs, categorized attending to the source from which they are obtained [5,6].

Among them, stem cells from human exfoliated deciduous teeth (SHEDs) have been isolated from the pulp of primary teeth [7]. Their multipotentiality has been confirmed in vitro by the expression of the mesenchymal lineage markers CD29, CD44, CD73, CD90, CD105, and CD146; the absence of expression of the hematopoietic lineage markers CD14, CD20, CD34, and CD45 [8,9,10]. Similarly, their osteo/odontogenic differentiation potential has been reported in various studies [11,12]. Altogether, the available evidence leans towards the possible application of SHEDs in tissue engineering and biologically based endodontic procedures [13].

Vital pulp treatment (VPT) procedures draw upon the reparative potential of the healthy and reversibly affected tissue from the dentin-pulp complex, aiming to preserve pulp vitality [14]. VPT for the primary dentition comprises various approaches, which vary in invasiveness: indirect pulp capping, direct pulp capping, and pulpotomy [15]. However, all of them are centered around the use of materials with specific biological properties to seal the viable tissue and provide a favorable medium for intrinsic repair [16]. 

Properties like cytocompatibility are expected from the biomaterials used for such purpose, meaning that when placed in direct contact with the cellular component of vital tissues, these will express physiological levels of proliferation, migration, and survival [17]. In addition, the materials used in VPT should exhibit bioactive properties, i.e., induce the formation of a mineralized hydroxyapatite-like attachment to the dentine substrate through the ionic interchange with tissue fluids in the process of biomineralization [18,19]. Lastly, biomaterials placed in contact with the dentin–pulp complex should ideally influence cell plasticity, inducing the osteo/odontogenic differentiation of local DSCs and, consequently, promote the process of tissue repair upon damage [20].

These properties are shared by a group of biomaterials, namely hydraulic calcium silicate-based endodontic cements (HCSCs) [21,22]. Available evidence reports their cytocompatibility, bioactive properties, and osteo/odontogenic differentiation induction in contact with human dental pulp stem cells (hDPSCs) from permanent teeth [23,24]. These characteristics resulted in the reception of HCSCs as suitable biomaterials for pulp capping in VPT procedures [14].

Considering the desirable properties expressed by HCSCs in contact with hDPSCs and the reported mesenchymal nature, multipotentiality, and osteo/odontogenic differentiation potential of SHEDs, it appears convenient to provide an updated review of their conjunct biological response for the potential use of different HCSC compositions in VPT on primary teeth. 

Accordingly, the present systematic review aimed to present a qualitative synthesis of the available literature consisting of in vitro assays, which assessed the cytocompatibility and bioactive properties of HCSCs in direct contact with SHEDs.

## 2. Materials and Methods

The present systematic review was performed in accordance with the Preferred Reporting Items for Systematic Reviews and Meta-Analyses (PRISMA) guidelines [25] and was registered in Open Science Framework (OSF) Registries (DOI: 10.17605/OSF.IO/H35ZE).

### 2.1. Criteria for Eligibility

In vitro studies evaluating the biological response of SHEDs to the treatment with HCSCs were eligible. Within the term biological response, assays assessing the cytocompatibility (i.e., cell viability, migration, proliferation), cell plasticity or differentiation (i.e., osteo/odontogenic marker expression), and bioactivity or biomineralization (i.e., mineralized nodule formation) were considered for inclusion. Assays comparing the previously mentioned variables both between two or more HCSCs and/or a control, and between a HCSC together with or without an additive were accepted. Studies assessing only one HCSC were also eligible. No inclusion/exclusion criteria were applied in terms of language or date of publication. The exclusion criteria were as follows: studies with methodological designs other than in vitro assays, studies assessing any DSC variant other than SHEDs, and/or studies assessing the biological behavior of dental biomaterials other than HCSCs or hydraulic calcium silicate sealers (HCSSs).

The inclusion criteria were established following the PICOS model [26], like so: Population/problem (P): stem cells from human exfoliated deciduous teeth; intervention (I): culture media conditioned with hydraulic calcium silicate-based cements; comparison/control (C): unconditioned culture media; outcome (O): cell viability, proliferation, migration, differentiation, and mineralization; study design (S): in vitro studies.

### 2.2. Search Terms and Strategy

The electronic database search, study selection process, variable extraction, and risk of bias analysis were performed by two independent researchers (J.L.S. and L.F.). In the case of any disagreement, a third examiner was consulted (F.J.R.-L). 

A systematic electronic database search was carried out in Medline, Scopus, Embase, Web of Science, and SciELO on March 31st and last updated on November 16th, 2020, without any language or year restrictions. The search strategy was devised taking into account previously published papers within the field of dental material research and their most cited descriptors. As a result, the following terms were selected: “silicate”, “bioceramic”, “stem cells from human exfoliated deciduous teeth”, “SHED, “cytocompatibility”, “biocompatibility”, “bioactivity”, “differentiation”, “expression”, and “mineralization.” “AND” and “OR” were used as Boolean operators to combine the search terms. The search strategy along with the search findings for the independent and combined search fields are shown in Table 1. In addition, the resulting study records were screened for additional potentially eligible studies.

### 2.3. Study Selection Process

Study records resulting from the search process were exported to Mendeley Desktop 1.19.4 reference manager software (Elsevier, AMS, The Netherlands) to manually check for duplicates. After discarding repeated records, reference titles and abstracts were screened following the previously mentioned criteria. Study records that did not meet any of the previously established inclusion criteria upon reading the title and abstract were discarded. Studies that met the criteria where then evaluated for eligibility for qualitative synthesis by full-text screening.

### 2.4. Data Extraction

Data extraction from the resulting studies was categorized as follows: variables for study characteristics, methodology, and results. Variables for study characteristics included authors and year of publication. Variables recorded for study methodology were, with regards to SHEDs, cell variant, cell passage, and donor age; regarding HCSCs, material/s used and its/their concentration; and with reference to the biological analyses, assays performed and their duration, and characteristics of the control groups used. Result variables recorded were the significant differences found for each assay, the time at which they were registered (duration), and their p value.

### 2.5. Quality Assessment (Risk of Bias)

Studies included in the present systematic review were independently assessed for inner methodological risk of bias by means of the “Modified CONSORT checklist of items for reporting in vitro studies of dental materials” [27], recording the fulfilment of each of the parameters or items considered in the checklist. Additionally, the percentage of item compliance of each of the studies was calculated.

## 3. Results

### 3.1. Search Results and Study Selection

**Figure 1:**
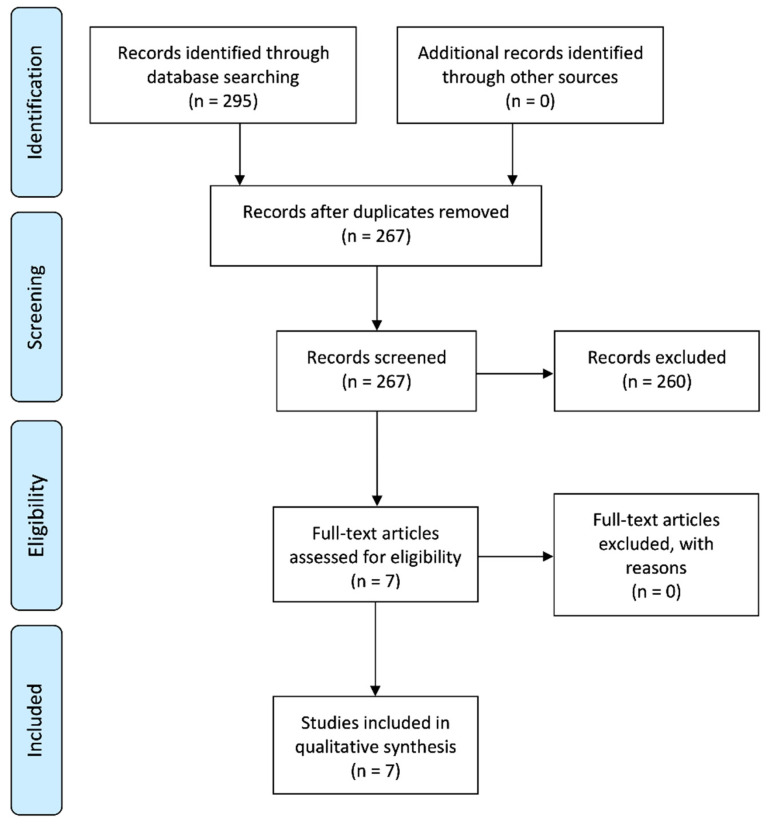
Systematic flow chart representing the study selection process. Based on the Preferred Reporting Items for Systematic Reviews and Meta-Analyses (PRISMA) flow diagram [26].

The search results and study selection process are illustrated in Figure 1. The electronic database searches identified 295 preliminary records: 178 from Scopus, 67 from Medline, 39 from Web of Science, and 11 from Embase. The search performed in SciELO database yielded no results. No additional eligible studies were found upon screening the references of the resulting studies. Duplicates were manually discarded by means of the reference manager software, resulting in 267 records. From these, 260 were excluded upon reading the title and abstract. The resulting seven papers were evaluated by full-text screening, and all of them were eligible for qualitative synthesis.

### 3.2. Study Methodology

Table 2 summarizes the methodology used by the included studies [28,29,30,31,32,33,34] to assess the viability, proliferation, migration, differentiation, and mineralized nodule formation of SHEDs treated with or without different concentrations of HCSCs.

The cells used for the in vitro biological assays performed by the studies included in the present review were SHEDs isolated from healthy donors, ranging from 3 to 12 years old. Specifically, studies generally selected cells at the 3rd to 6th passages for the analyses, with the lowest being cells at the 2nd passage [30] and the highest at the 8th passage [31].

The modal HCSCs assessed were Biodentine (BD; Septodont, Saint-Maur-des-Fosses, France), used by five studies [28,30,31,32,34], and Mineral Trioxide Aggregate (MTA; Angelus, Londrina, PR, Brazil), used by four studies [28,29,31,34]. The remaining HCSCs considered in the present review were evaluated by one in vitro study, as follows: ProRoot MTA (PR MTA; Dentsply Tulsa, TN, USA), [33] and iRoot BP Plus (iRP; Innovative BioCeramix Inc., Vancouver, BC, Canada), [29]. A resin-modified calcium silicate biomaterial (Theracal LC (TLC); Bisco Inc., Schamburg, IL, USA) was assessed and compared with HCSCs in one study [34] and was consequently included in our qualitative synthesis.

With reference to the cytocompatibility analyses, a wide range of assays were performed by the included studies. To assess cell viability, the modal assay performed was the 3-(4,5-dimethylthiazol-2-yl)-2,5-diphenyltetrazolium bromide (MTT) assay [28,30,31,32,34]. Other methods used to evaluate cell viability were the Cell Counting Kit-8 or CCK8 [29] and live/dead fluorescent staining [30]. A total of five studies assessed cell migration using either wound healing, transwell migration, or similar assays [29,31,32,33,34]. Among them, two studies used Annexin-V/7-AAD staining to measure cell apoptosis [33,34]. Lastly, cell adhesion and/or morphology was assessed under scanning electron microscopy (SEM) or by immunofluorescence staining [29,30,32,34].

As to bioactivity analyses, the majority of studies used Alizarin Red Staining (ARS) to evaluate the mineralization potential of HCSC-treated SHEDs. [28,29,30,34]. Two studies used quantitative reverse transcriptase polymerase chain reaction (RT-qPCR) to assess the differentiation of HCSC-treated SHEDs [30,31], and one study performed an alkaline phosphatase (ALP) assay to evaluate cellular activity [29].

### 3.3. Study Results

The significant results reported by the included studies for the aforementioned cytocompatibility and bioactivity assays are presented in Table 3 and Table 4, respectively, along with their significance level (*p* value).

Cell viability assays revealed significant positive results for MTA [28,29,31,34], BD [28,32,34], and iRP [28] treatment when compared to a negative control group (SHEDs cultured in unconditioned medium), whereas SHED culture with TLC exhibited a significantly lower cell viability than the negative control [34]. SHED treatment with BD reported a significantly higher cell viability than MTA in two studies [28,34], while the opposite was observed in another study [31]. In a similar manner, treatment with iRP produced significantly higher cell viability and migration rates when compared to MTA, but both of them were significantly higher than the negative control [29].

Regarding cell migration assays, a significantly higher cell migration was observed in both the treatment of SHEDs with MTA [28,29,31,34] and BD [28,31,32,34] when compared to a negative control. SHED culture with iRP also produced a significantly higher cell migration [29]. Similar to the cell viability assays, the treatment with TLC reported significant negative results in terms of SHED migration [34]. Both the treatment with BD and MTA resulted in similar cell migration rates, except in one study, in which BD showed significantly higher SHED migration using a sulforhodamine B (SRB) assay [31]. In the same study, both BD and MTA showed significantly lower cell viability and migration rates when compared to a positive control (SHEDs cultured with a 20% fetal bovine serum (FBS) supplement). 

Bioactivity assays using ARS to assess SHED mineralization potential revealed positive significant results for MTA [28,29], BD [30,34], and iRP [29], compared to a negative control. In addition, both BD [28] and iRP [29] showed a significantly higher mineralization potential than MTA. Furthermore, iRP showed significantly higher ALP activity than MTA, both of them higher than the control group [29]. SHEDs treated with TLC, consistent with the results shown in the cytocompatibility assays, exhibited a significantly lower mineralization potential than the negative control group [34].

### 3.4. Quality Assessment (Risk of Bias)

The results of the quality assessment using the previously mentioned modified CONSORT checklist are presented in Table 5. The mean item compliance of the included studies was 60%, with a maximum score of 64%, and a minimum score of 57%. Items 5–9 and 14 regarding sample size calculation, randomization process, and availability of a study protocol were not fulfilled by any of the studies. Items 2, 3, 4, 10, 11, and 13 regarding the description of the methodology, statistical analysis and significance, and funding data were fulfilled by all of the included studies.

## 4. Discussion

The evaluation of the biological properties of HCSCs and other available pulp capping biomaterials towards dental pulp cells from the permanent dentition or hDPSCs has been investigated by various in vitro studies [24,35,36,37] and recently reviewed from different methodological perspectives [17,38]. Altogether, the evidence highlights the cytocompatibility and bioactive nature of HCSCs towards HDPSCs. Additionally, various clinical trials assessing the effectiveness of HCSCs in VPT for the treatment of carious pulp exposures in the permanent dentition with reversible pulpitis report high success rates after variable follow-up periods [39,40]. 

Most recently, the implementation of this group of biomaterials in biologically based procedures for the primary dentition has been investigated, focusing on the biological response of the cellular component of the primary pulp tissue towards them. Thus, the aim of the present systematic review was to perform a qualitative synthesis of available evidence on the in vitro cytocompatibility and bioactivity of HCSCs towards SHEDs, providing an updated and structured analysis of the current knowledge with regards to this framework. 

After the systematic search strategy and selection process, a total of seven studies met the previously established inclusion criteria and were included for the qualitative synthesis. Albeit limited, the study sample comprised an extensive range of cell viability, migration, proliferation, adhesion, and bioactivity assays regarding the biological response of SHEDs towards four different commercially available HCSCs (MTA, PR MTA, BD, iRP) and a resin-based silicate-based biomaterial (TLC). 

In general terms, the in vitro biological assays considered in the present review were performed by culturing SHEDs with variable HCSC dilutions for specific time periods in standardized conditions, reporting a series of outcome variables with a negative and/or positive control group as a reference. The characteristics of the groups used as a control were specified by all of the included studies, as shown in Table 2. The majority of studies presented the results of the different cytocompatibility and bioactivity assays using only a negative control group as a reference [29,32,34], while the remaining studies used both a negative and a positive control group as a reference [28,30,31].

Negative control groups consisted of SHEDs incubated in unconditioned culture media. Alpha minimum essential medium (α-MEM) with a series of supplements was used by all of the studies, except in two cases, in which Dulbecco’s modified Eagle medium (DMEM) was used [28,34]. Supplements used included fetal bovine serum (FBS) at different concentrations (10% [28,29,31] or 15% [30,33]), antibiotic (penicillin, streptomycin, and/or amphotericin) solutions [28,29,30,31,33,34], L-glutamine [28,33], and L-ascorbic acid phosphate [30,33]. The positive control groups used differed in their composition, varying from the use of an osteoinductive medium [28], to the use of the negative control medium plus a series of supplements: 20% FBS [31], dexamethasone disodium phosphate, KH2PO4, and b-glycerophosphate [30]. The varying characteristics of the culture media may hinder the validity of the analyses and comparisons of the reported outcomes, highlighting the importance of the standardization of the protocols used by future studies in the field. It may be worth mentioning that a series of guidelines developed by the International Organization for Standardization with regards to the sample preparation and in vitro evaluation of cytotoxicity (ISO 10993-12:2012(E) and ISO 10993-5:2009(E), respectively) are currently available as a reference.

The methodological heterogeneity among the included studies resulted in a wide variety of outcomes. Nonetheless, as shown in Table 3, significant results from SHED viability and migration assays tended to support the treatment with BD, MTA, and iRP as opposed to the culture in unconditioned media. The same was observed from the bioactivity assays (Table 4), in which the treatment with all the previously mentioned HCSCs resulted in a significantly higher mineralized nodule formation and/or ALP activity than the negative control groups. Regarding the differences between the studied HCSCs, the limited number of comparisons added to the similar results shown by all the tested biomaterials results in insufficient evidence to support the use of one specific HCSC. Collectively, these results are consistent with the results reported from available literature on the use of HCSCs in VPT for the primary dentition [41,42,43,44]. Specifically, recent systematic reviews of randomized controlled trials (RCTs) have reported high success rates of VPT procedures with MTA and BD on primary teeth with varying degrees of pulp inflammatory states, without significant differences between them [45,46]. 

On the other hand, TLC, a resin-modified calcium silicate-filled biomaterial, showed significant negative results in both cytocompatibility and bioactivity assays when cultured with SHEDs. Interestingly, these results are also consistent with results from a recent RCT, in which both MTA and BD showed a superior performance than TLC as partial pulpotomy agents [47]. However, as a direct pulp capping agent, TLC exhibited a comparable outcome to MTA for the treatment of primary molars in a different RCT [48]. As a result, the evidence regarding the biological properties of TLC towards primary pulp cells and tissue could be categorized as inconsistent, requiring further research on the use of TLC in VPT procedures for the primary dentition.

Various descriptive assays were also performed by the included studies, including the evaluation of SHED morphology and adhesion under SEM [30,32,34]. Despite no statistical significance being reported from these assays, their results indicated a positive biological response of SHEDs to the treatment with BD, MTA, and iRP, and a negative response towards TLC, as observed in the cytocompatibility and bioactivity assays.

Contrary to the general tendency, the study assessing the cytocompatibility of PR MTA towards SHEDs [33] reported a decreased cell viability and increased cell apoptosis after a direct contact with 1 week post-set PR MTA. As highlighted by the authors, the majority of similar in vitro studies assess the biological properties of material eluates [30,34], although the cellular response to the materials may depend on the use of fresh or cured materials, direct contact or extracts of the materials, and the concentration of the materials in the culture media [49]. Consequently, the results shown in the aforementioned study highlight the need for the use of a uniform methodology in different experimental conditions in order to comprehensively assess the biological properties of HCSCs.

As shown in the methodological summary (Table 2), different HCSC concentrations were assessed among the included studies. In all cases, material preparation was performed following the respective manufacturers’ instructions. However, with regards to the concentration used, studies followed several routes. In various cases [28,31,32], material dosage was selected based on previous works [50,51,52,53,54], while others followed the respective ISO standards for sample preparation [30,34], and assessed a series of material dilutions.

Thus, various concentrations were assessed for MTA, BD, iRP, and TLC. Those that exhibited positive significant results in SHED cytocompatibility and bioactivity assays using a negative control as a reference were: for MTA, 1mg/mL [28,31]; for BD, 1mg/mL [28,31] and 0.02, 0.2, and 2mg/mL [32]. These dosages appear as potentially optimal in terms of the biological response of SHEDs in vitro and may be of use as a reference in future studies in the field. Additionally, the biological effect of HCSCs on various types of DSCs has been reported as dose-dependent [30,55], so the evaluation of the biological behavior of SHEDs towards a wider range of biomaterial concentrations could be a practical line of research.

Similarly, the role of HCSCs on SHED osteo/odontogenic differentiation should be further explored. From the included studies, the analysis of osteo/odontogenic marker expression was only assessed twice [30,31], by means of RT-qPCR. In the first study, the expression of dentin matrix protein-1 (DMP-1) by SHEDs after the treatment with 1mg/mL MTA or BD was evaluated, observing an upregulation of such a marker when compared to a negative control in a 21 day culture period. In the second case, a series of BD dilutions were tested for the expression of a various osteo/odontogenic markers, reporting a concentration dependence of the biological effects of this HCSC. A similar pattern has been described from the biological response of hDPSCs towards different HCSCs in various studies, exhibiting a significant upregulation of a wider variety of osteo/odontogenic markers: dentin sialophosphoprotein (DSPP), osteocalcin (OCN), osteopontin (OPN), ALP, DMP-1, Runt-related transcription factor 2 (Runx-2), bone sialoprotein (BSP), among others [53,56,57,58]. In order to increase the validity of the conclusions reached with reference to the influence of HCSCs on SHED plasticity and, specifically, osteo/odontogenic differentiation, further investigations are necessary.

To the authors’ knowledge, this is the first systematic review to assess the in vitro biological response of SHEDs to the treatment with HCSCs. Previous systematic reviews have assessed the biological in vitro properties of HCSCs towards human tooth pulp cells [38] and towards specific types of DSCs, namely human dental pulp stem cells (hDPSCs) [17] and human stem cells from the apical papilla (hSCAPs) [59]. DSCs, as a subfamily of precursor cells, share a mesenchymal nature but have shown individual properties, which could result in diverse responses to the influence of external factors. For instance, studies have reported that DSCs from the periodontal ligament (periodontal ligament stem cells or PDLSCs) have shown a higher osteogenic differentiation potential than hDPSCs [60] and SHEDs [61]. Additionally, previous reviews regarding the different DSC variants have acknowledged their differences. SHEDs have shown a higher proliferation rate than DPSCs and have been in fact categorized by some studies as “immature DPSCs” or iDPSCs, thereby highlighting their differences. Furthermore, each DSC variant expresses specific phenotypic characteristics in terms of marker expression [6,62].

Altogether, the differences between DSCs act as a justification for the need for the individual evaluation of the biological response of dental biomaterials to the different DSC variants identified [5,6], as performed by all of the in vitro studies included in the sample and by the present review with SHEDs. 

As previously observed in the aforementioned reviews for other DSCs, SHEDs generally exhibited adequate levels of cell viability, proliferation, migration, and an increased mineralized nodule formation after incubation with various calcium silicate-based compositions, acting as supporting evidence for their potential use in biologically based endodontic procedures.

## 5. Conclusions

The results from in vitro assays assessing the viability, proliferation, migration, differentiation, and mineralization potential of SHEDs treated with the HCSCs MTA, Biodentine, and iRoot BP Plus highlight their adequate cytocompatibility and bioactive properties, supporting their use in VPT procedures for the primary dentition. However, evidence in this regard remains limited, and critical aspects such as influence of this group of biomaterials on SHED plasticity and osteo/odontogenic differentiation potential should be further explored in order to increase the predictability of their biological behavior in the clinical setting.

## Figures and Tables

**Table 1:** Search strategy and findings per database.

Database	Search Strategy	Findings
Medline	#1 (silicate) OR (bioceramic)	47,275
	#2 (stem cells from human exfoliated deciduous teeth) OR (SHED)	60,545
	#3 (((((cytocompatibility) OR (biocompatibility)) OR (bioactivity)) OR (differentiation)) OR (expression)) OR (mineralization)	4,715,994
	#1 AND #2 AND #3	67
Scopus	#1 ALL (silicate OR bioceramic)	508,929
	#2 ALL (stem cells from human exfoliated deciduous teeth OR SHED)	2972
	#3 ALL (cytocompatibility OR biocompatibility OR bioactivity OR differentiation OR expression OR mineralization)	9,035,995
	#1 AND #2 AND #3	178
Embase	#1 (silicate OR bioceramic)	24,472
	#2 (stem cells from human exfoliated deciduous teeth OR SHED)	72,196
	#3 (cytocompatibility OR biocompatibility OR bioactivity OR differentiation OR expression OR mineralization)	4,096,064
	#1 AND #2 AND #3	11
Web of Science	#1 TS = (silicate OR bioceramic)	108,951
	#2 TS = (stem cells from human exfoliated deciduous teeth OR SHED)	185,496
	#3 TS = (cytocompatibility OR biocompatibility OR bioactivity OR differentiation OR expression OR mineralization)	4,004,895
	#1 AND #2 AND #3	39
SciELO	#1 (silicate OR bioceramic)	675
	#2 (stem cells from human exfoliated deciduous teeth OR SHED)	12
	#3 (cytocompatibility OR biocompatibility OR bioactivity OR differentiation OR expression OR mineralization)	21,081
	#1 AND #2 AND #3	0

**Table 2:** Summary of the methodology of the included studies.

Author	Cell Variant	Materials Used	Control Groups	Activity Analysis	Duration
Dahake et al., 2020 [28]	SHED (8–12 years), 5th passage	MTA (1 mg/mL), BD (1 mg/mL),	Negative: SHED + DMEM, 10% FBS, 2 mmol/L L-glutamine, 1% penicillin, streptomycin, and amphotericin (PSA); Positive: SHED + DMEM, 20% FBS, 50 μg/mL ascorbic acid, 50*n* mol/L β glycerol phosphate, 10–8 mol/L dexamethasone.	MTT Assay	7 days
				ARS	14 days
Wang et al., 2019 [29]	SHED (6–10 years), 4–6th passage	iRoot BP (n/s), MTA (n/s)	Negative: SHED + αMEM, 10% FBS, 100 U/mL penicillin, 100 mg/mL streptomycin.	CCK8	1,3,5,7 days
				Transwell migration assay	24 h
				Wound healing assay	24 h
				Immunofluorescence staining	1,3,5 days
				ALP activity assay	7,14 days
				ARS	21 days
Athanasiadou et al., 2018 [30]	SHED (3–10 years), 2–6th passage	BD (1:1, 1:2, 1:4, 1:8, 1:16, 1:32, 1:64, 1:128 eluates)	Negative: SHED + αMEM, 15% FBS, 100 μM L-ascorbic acid phosphate, 100 U/mL penicillin, 100 mg/mL streptomycin, 0.25 mg/mL Amphotericin B; Positive: negative + 0.01 mM dexamethasone disodium phosphate, 1.8 mM KH_2_PO_4_, 5 mM b-glycerophosphate.	MTT Assay	24, 72, 120 h
				SEM	72 h
				Live/dead fluorescent staining	72 h
				qRT-PCR	7,14 days
				ARS	14 days
Araújo et al., 2018 [31]	SHED (7–8 years), 4–8th passage	MTA (1 mg/mL), BD (1 mg/mL),	Negative: SHED + αMEM, 10%FBS, 1% penicillin-streptomycin; Positive: SHED + αMEM, 20% FBS, 1% penicillin-streptomycin.	MTT assay	1,3,5,7 days
				SRB assay	1,3,5,7days
				Cell migration assay	Overnight
				qRT-PCR	1,7,14,21days
Awidi et al., 2018 [32]	SHED (5–6 years), 3rd passage	BD (0.02 mg/mL, 0.2 mg/mL, 2 mg/mL, 20 mg/mL)	Negative: SHED + αMEM, 5% platelet lysate.	MTT assay	6 days
				Wound healing assay	24 h
				Transwell migration assay	24 h
				Cell adhesion assay	1 h
Tsai et al., 2018 [33]	SHED (5–7 years), 3–4th passage	1g PR MTA:5 ml dd H_2_O	Negative: SHED + αMEM, 15% FBS, 100 μM L-ascorbic acid phosphate, 2 mM L-glutamine, 100 U of antibiotic-antimycotic.	Wst-1 assay	1,2,3 days
				Annexin-V/7-AAD staining	2 days
Collado-González et al., 2017 [34]	SHED (6–9 years), n/s	1:1, 1:2 and 1:4 of: BD, MTA, Theracal, IRM	Negative: DMEM + penicillin-streptomycin.	MTT assay	24,48,72 h
				Annexin-V/7-AAD staining	72h
				Cell migration assay	24,48 h
				SEM	3 days
				ARS	7,14,21 days

h: hours; n/s: not specified. BD: Biodentine, MTA: MTA Angelus, PR MTA: ProRoot MTA.

**Table 3:** Significant results in cytocompatibility assays.

Author	Assay	Significant Results	Duration	*p* Value
Dahake et al., 2020 [28]	MTT assay	BD > MTA > -control	7 days	*p* < 0.001
Wang et al., 2019 [29]	CCK8	iRoot BP, MTA > -control	7 days	*p* < 0.05
		iRoot BP > MTA > -control	3,5 days	*p* < 0.05
	Transwell migration assay	iRoot BP > MTA > -control	24 h	*p* < 0.05
Athanasiadou et al., 2018 [30]	MTT assay	1:16, 1:32, 1:64 BD > -control	72 h	*p* < 0.05
Araújo et al., 2018 [31]	MTT assay	+control > MTA, BD, -control	3,5 days	*p* <0.05
		MTA > BD, -control	7 days	*p* < 0.05
	SRB assay	+control > MTA, BD	1,3,5,7 days	*p* < 0.05
		BD > MTA	3,5 days	*p* < 0.05
	Cell migration assay	BD, MTA > -control	Overnight	*p* < 0.005
Awidi et al., 2018 [32]	MTT assay	0.02, 0.2, 2 mg/mL > 20 mg/mL	6 days	*p* < 0.0001
	Transwell migration assay	0.02, 0.2, 2 mg/mL > -control	24 h	*p* < 0.0037
Tsai et al., 2018 [33]	Wst-1 assay	-control DC > PR MTA DC	1 days	*p* < 0.0001
			2 days	*p* < 0.01
			3 days	*p* < 0.05
		-control IDC > PR MTA IDC	1 days	*p* < 0.05
			3 days	*p* < 0.01
Collado-González et al., 2017 [34]	MTT assay	MTA > -control	48,72 h	*p* < 0.01
		BD > -control	48,72 h	*p* < 0.001
		BD > MTA	48,72 h	*p* < 0.01
		-control > Theracal	24,48,72 h	*p* < 0.001
	Cell migration assay	BD > -control	48 h	*p* < 0.001
		MTA > -control	48 h	*p* < 0.001
		-control > Theracal	48 h	*p* < 0.001

h: hours. -control: negative control.

**Table 4:** Significant results in bioactivity assays.

Author	Assay	Significant Results	Duration	*p* Value
Dahake et al., 2020 [28]	ARS	+control > BD > MTA > -control	14 days	*p* < 0.001
Wang et al., 2019 [29]	ALPs	iRoot BP > MTA > -control	7,14 days	*p* < 0.05
	ARS	iRoot BP > MTA > -control	21 days	*p* < 0.05
Athanasiadou et al., 2018 [30]	ARS	1:4, 1:8, 1:16, 1:32 BD > -control; +control > 1:1, 1:2, 1:4, 1:8, 1:16 BD	14 days	*p* < 0.05
Collado-González et al., 2017 [34]	ARS	BD > -control	7 days	*p* < 0.01
			14 days	*p* < 0.05
			21 days	*p* < 0.001
		-control > Theracal	7, 21 days	*p* < 0.01
			14 days	*p* < 0.001

h: hours. -control: negative control.

**Table 5:** Quality assessment results.

Studies	Modified CONSORT Checklist															
	1	2a	2b	3	4	5	6	7	8	9	10	11	12	13	14	%
Dahake et al., 2020 [28]	Y	Y	Y	Y	Y	N	N	N	N	N	Y	Y	N	Y	N	57
Wang et al., 2019 [29]	Y	Y	Y	Y	Y	N	N	N	N	N	Y	Y	Y	Y	N	64
Athanasiadou et al., 2018 [30]	Y	Y	Y	Y	Y	N	N	N	N	N	Y	Y	Y	Y	N	64
Araújo et al., 2018 [31]	Y	Y	Y	Y	Y	N	N	N	N	N	Y	Y	Y	Y	N	64
Awidi et al., 2018 [32]	Y	Y	Y	Y	Y	N	N	N	N	N	Y	Y	N	Y	N	57
Tsai et al., 2018 [33]	Y	Y	Y	Y	Y	N	N	N	N	N	Y	Y	N	Y	N	57
Collado-González et al., 2017 [34]	Y	Y	Y	Y	Y	N	N	N	N	N	Y	Y	N	Y	N	57

Y: reported on the article; N: not reported on the article; %: percentage of compliance per article. Based on the checklist from “Guidelines for Reporting Pre-clinical In Vitro Studies on Dental Materials” [27].
